# Supplementary material for: PandoGen: Generating complete instances of future SARS-CoV-2 sequences using Deep Learning
Source: PLoS Comput Biol. 2024 Jan 19;20(1):e1011790. doi: 10.1371/journal.pcbi.1011790 (PMC10829978; doi:10.1371/journal.pcbi.1011790)
Supplement: Attachment — Submitted filename: ResponseToReviewers.pdf [file pcbi.1011790.s002.pdf]

We want to thank the reviewers for their insightful comments. We believe that addressing these comments has improved our manuscript significantly. Below, we outline the steps we have taken to address the comments.

Author Responses to Reviewer 1

Reviewer Comment: *Overall, I think the authors have presented a well-structured pipeline in generating novel sequences with good predictability. The use of a reward model to predict potential infectiousness is innovative. The objective function combining a MLE for sequence prediction and a penalty term to limit deviation from the SARS-CoV2 spike protein sequence is very interpretable. And their results has shown capability to produce novel sequences that later occurred as SARS-CoV2 sequences. I believe the authors have presented sufficient results to show that PandoGen is capable of producing sequences that are not in the training set. And they have demonstrated predictability of the model both in fig2 and in fig3 with the improved PPV results when considering high rank sequences. Additionally, it is nice to see Pandogen's PPV score improves with a stricter top-n sequence ranking threshold as it shows efficacy of the ranking measures.*

Author Response: We thank the reviewer for the encouraging feedback.

Reviewer Comment: *In terms of presentation of the methods and results, I think readability and accessibility could be improved.*
For example:

Since the performance evaluation metrics employed in this work are proposed by the author, it would be helpful if the evaluation measures were clearly defined and stated.

Author Response: We have added detailed explanations regarding the metrics used in the evaluation in Section 2.1.3.

Reviewer Comment: *Another thing is the reward system is mentioned early in the manuscript but only explained further in section 5.*

Author Response: We have added more detailed explanations at the start of the manuscript in Section 1. We believe that now, Sections 1 and 2 describe the methods sufficiently well for readability, and Section 4 (previously Section 5) can be referenced for details on reproducibility.

Reviewer Comment: *I would also like to suggest mentioning of the length of the sequences used for the analysis since it impacts the computation costs a lot.*

Author Response: We have added this information in the manuscript in Section 2.1.3.

Reviewer Comment:

I am curious about the number of sequences generated to obtain the top-ranking sequences, especially considering the improvement from around 30% PPV in Figure 2 to approximately 20%-70% PPV in Figure 3.

Moreover, since the methods used for comparison produce significantly fewer novel sequences, I find the evidence supporting 'better performance in predictability' not very strong. Please discuss why ~30% predictability (PPV) should be considered a "good result".

Relatedly, in figure 2, the methods used for comparison to the proposed PandoGen rarely produce novel sequences. I think that explains why Prot GPT2 enumerated is showing a big variance in the PPV plot (due to a very small denominator). In my opinion, if all methods are producing a lot of novel sequences, but PandoGen is dominating PPV then it would be more convincing to claim PandoGen performs better in terms of novel sequence prediction.

Author Response: We apologize for the confusion caused by our presentation. We have addressed these points in the manuscript. We offer a brief explanation below.

The number of sequences used for quantitative analysis in Figures 2 and 3 are the same. They are plots of the same data – in one case, we take the cumulative stats for the data, and in the other, we plot them against sequence rank.

As the reviewer correctly noted, there were operating points for one of the competing methods ("Prot GPT2 enumerated") where it was producing very few novel sequences resulting in very wide variance for those operating points for that method. To clean the data better, we removed operating points for all methods where 99% or more of their output samples were simply reproductions of the training set (i.e. less than 1% novel sequences). In addition, for "Prot GPT2 enumerated", we noticed that the method wasn't producing a sufficient number of novel sequences across its normal operating range, and hence didn't have sampling configurations that produced similar number of novel sequences as PandoGen's best operating points. To account for this, we added new operating points for that method based on temperature shaping, a technique used to improve output variety in LLMs, that produce comparable number of novel sequences to PandoGen.

Previously, Figure 2 plotted output sample statistics against an operating point parameter of the model on the X-axis. This made it difficult to compare the methods as the operating point parameter is not universally interpreted by the models in the same way. To avoid this confusion, we have changed Figure 2 to be plotted against the number of novel sequences in the generated sample from each method on the X-axis. This shows clearly that when methods are producing similar numbers of novel sequences, PandoGen outperforms competitors.

Finally, we want to emphasize that PPV is only one aspect of the output samples from the model. The quality of the output sequences also depends on the infectiousness of the generated sequences, and the number of novel lineages forecasted by the generated samples. Even if a model generates samples with 100% PPV, if the generated sequences account for very few infectious cases, and produce no new lineage forecasts, the output sample is not very valuable. We have added more material – both data and explanations - to emphasize the significance of these additional relevant measurements.

We believe that the new data along with corrections to the plots highlight PandoGen's ability to generate samples with higher predictivity, producing sequences with higher infectiousness potential, and covering significantly more novel lineages than competing methods.

Author Responses to Reviewer 2

Reviewer Comment: *The authors of this paper have achieved an impressive feat of training an AI to predict future sequences of the SARS-CoV-2 virus. The work appears to be mostly methodologically sound. However, the current version of the manuscript is heavy going, but key methodological details and results are left lacking, as is a robust discussion of how this work relates to existing AI models already published in some landmark studies. Whilst the authors mention other works, their discussion of these and what is novel about their work is lacking, it's hard to evidence originality with the manuscript in it's current form.*

Furthermore, they don't robustly compare their work to these other methods, focusing on a single competing methodology only. There are also issues of the biggest claims made by the work being presented in rather vague terms, with the details buried within supplemental information or a terse methods section, which feels off and like an effort to evade scrutiny, clearly more work needs to be done to prove that that methods are robust and I highlight a few key areas where more discussion is needed of shortcomings, and I'm especially concerned about the suitability of the PANGO lineage classifications used from GISAID. The work is rather lacking in biologically meaningful conclusions, feeling rather like an incremental improvement on prior works. I also can't find any mention of code availability, which is troubling. I believe that with a reworked manuscript, this work could potentially be acceptable for publication; I outline the issues I would like to see addressed below:

Author Response: We acknowledge the important points raised by the reviewer. We thank the reviewer for identifying the following shortcomings in our manuscript:

- Unclear presentation of methods.
- Unclear comparison of our method to other methods, and lack of discussion of how our method relates to existing AI models.
- Unclear explanation of the main claims of the paper including biological relevance of the results and the novelty of our work.
- Missing Code availability.

We have taken the following broad actions, which we will address in more detail below.

First, we have streamlined the presentation of the methods, adding plenty of details, which we believe are substantially more readable and clearer in Section 1. We have written these parts with the intention that the Methodology section needs to be read primarily for finer details of the methods, and the main sections – Introduction and Results – clearly describe the main points of our methodology and novelty.

Second, we have removed the prior Related Works section, and integrated that material closely into the explanation of our work in Section 1. Now, Section 1 explains how our method relates to

existing AI models, as well as the novelty of our work. Specifically, it explains what we do that prior work doesn't do, and what type of experiments and benchmarking we perform that is not performed in prior work. We also explain how we chose competing methods and our reasons for these selections in Section 2.

Third, we have clearly and precisely listed our main results in Section 1, as well as in Section 2. We have also explained the biological significance of the sequences produced by our model and included specific comparisons to highlight these aspects in Sections 1 and 2. We have explicitly presented the quantities that our method improves upon comparison to competing approaches.

Finally, we have included a separate code section explaining the code availability, and we have enhanced the user guide in our code repository.

Reviewer Comment: *The canonical name for the virus that causes COVID-19 is SARS-CoV-2 and not "SARS-CoV2", as used throughout the manuscript. This should be corrected to reflect the current literature.*

Author Response: We have corrected this in the manuscript.

Reviewer Comment: *The authors use a discriminative model to train the AI for an "infectiousness potential", using pairwise competitions between sequences which are temporally close within the pandemic. The authors do state that there are issues with inferring this from data within GISAID alone, and I believe the authors have approximated the first order of this problem successfully using occurrence data alone, and they appear to use robust heuristics for the comparisons. However, there are issues here in that, potentially, one sequence is conferred an advantage over another due to factors not captured within their model. Such a viral sequence first occurring at a superspreader event or within a transport hub of a densely populated city would have a much more significant "founder effect" than a new viral sequence first occurring in a remote location. While it is potentially challenging to model this effect, it would be good for the authors to more thoroughly acknowledge the limitations in their works and that going off the frequency of sequence observation alone is not a perfect solution. I would like a more detailed discussion of the founder effect within the main body of the manuscript and how this confounds their "infectiousness" potential.*

Author Response: We use sequence information, reporting dates, and geographic locations to shortlist sequence comparisons that are likely to be informative about sequence infectiousness. Specifically, for comparing two sequences for training our reward model, (1) we need the sequences to be first reported close to each other in time (2) we need sequence count ratios to be similar in two disjoint geographic regions and (3) we do not use information regarding new sequences first reported in the last 7-week period of the training period to train the reward model. However, as the reviewer correctly points out, there are confounding factors such as superspreader events, and transport hubs which may skew these numbers in favor of some sequences over others through factors other than infectiousness. As such, our approach has the following limitations: (1) some data from the most recent weeks are not used to train the reward model (though it is used to train the generative model), which can result in our model not responding to certain sudden developments (2) our heuristics for ensuring fairness use sequence

metadata which does not include information on specific situations in different locations or times (e.g., local events, travel patterns etc.), and hence some short-term events or other locality-specific characteristics can still contaminate the data. A more thorough analysis of locations and times by integrating external knowledge such as nature of location (e.g., “transport hub”), or special events taking place in different locations (e.g., “conference” or “festival”) can improve on our assumptions further. The exact form of these analyses is not clear to us, but they are worthy of future study.

We have clearly acknowledged the limitations of our approach and presented future research directions in Section 4.5.

Reviewer Comment: *In line 165, the authors state that PandoGen forecasts many of these variants ahead of time; they need to give more details here for a claim like this.*

Author Response: As part of our rewrite of the Introduction, this claim has been made clearer in Section 1, and Section 2.2.

Reviewer Comment: *Line 312 specifically this statement: “sequences with 10 or more cases constitute less than 10% of all sequences.” this whole concept needs better explanation: do they mean 10 or more cases in their predicted sequences? The authors’ concept of what constitutes a “salient” sequence needs a better description so as not to confuse the reader, especially since this is a key concept of the manuscript.*

Author Response: Salient sequences are sequences with 10 or more cases in GISAID. Salient sequences are biologically significant because they indicate a higher likelihood for infectiousness. Less than 10% of sequences in GISAID are reported 10 times or more in GISAID. Hence being able to produce more salient sequences indicates a method’s ability to zoom in on the more potent viral sequences. We have made this explicit and clear in our descriptions. Section 1, and Section 2.1.3 define salient sequences. Figures 3, and 7 benchmark the ability of different models to generate salient sequences.

Reviewer Comment: *On line 341, the authors state: “PandoGen predicts a significant proportion of all novel lineages reported for many weeks after training.” This and the following line is too vague a claim. They need to be more specific when discussing the performance of their methods; specifically, which time point do they mean here?*

Author Response: We have made this claim clearer, precisely describing the timeline and the fraction of lineages. The description has been updated in Section 2.1.3.

Reviewer Comment: *Line 353, the comment “We generate and analyze samples to see whether important pandemic variants such as Delta and Omicron sublineages can be consistently generated by PandoGen” about the ability to generate the sequences of Delta and Omicron requires much more detail. This is potentially a significant finding, but doesn't seem fully supported.*

Author Response: We thank the reviewer for highlighting this. We agree those are important outcomes of our modeling effort and should be treated thoroughly. The sentences highlighted by the reviewer were previously presented in Section 2.1 without additional context. We describe experiments that support these claims in Section 2.2 with details on training data, sampling from the model, and experimental evaluation. As a result, the original sentences are no longer necessary, and have been removed.

Reviewer Comment: *In lines 359-362, the authors compare their method to that of Prot GPT 2. However, all details are buried within the supplemental; a summary of these findings must be present within the main body of the manuscript. Equally, should a comparison to other methods not be made at this point too?*

Author Response: In the new manuscript, we have added one more representative method ProGen2, which is the largest PLM-based *de novo* protein generator in literature. The main comparisons among the three methods: PandoGen (ours), Prot GPT2, and ProGen2 are in lines 443 to 561 in the main manuscript (Section 2.1.3). These are supported quantitatively by Figures 3 – 7 also in the main manuscript. There are some auxiliary comparisons in the Supplementary Document.

Reviewer Comment: *Around line 386, the authors discuss using data within GISAID, specifically the Pango lineage, as training data. However, can we depend on GISAID assignments here? Would it not be better to download and fully reassign lineages to all GISAID sequences? Because until PANGO defines a lineage, it can't be assigned by pangolin? Thus, the validity of the whole study here is predicated on the assumption that GISAID respectively updates its lineage assignments, and I can't find any documentation stating they do! Potentially, they may have trained their models naively on sequences that GISAID missed, as the PANGO definitions for Delta and Omicron have not yet been released or updated such that GISAID could not classify these early sequences as such. This point needs to be cleared up as the whole study is based on an assumption of arcuate lineage assignment within GISAID, is this re-visited and updated by GISAID, or is it only performed once at sequence submission time?*

Author Response: We agree that the reviewer's comments on lineage integrity in GISAID need to be addressed. We had assumed that GISAID consistently updates PANGO lineages without direct confirmation, and hence verifying whether this is the case is important. Following the reviewer's advice, we downloaded the latest set of genomic sequences from GISAID and performed lineage assignment using the Pangolin tool's latest release (v4.3.1).

The new Pangolin lineage assignment agreed with the GISAID lineage assignment in our experiment data file in the case of 95% of Accession IDs. When looking only at unique Spike protein sequences, the agreement was over 86%. The numbers are respectively 97.8% and 90.5% when looking only at sequences used as training data. More details are in the manuscript Section 4.1. as well as in the Supplementary Document.

On the evaluation side, all evaluations in our manuscript have been changed to the new lineage assignments that we obtained from running Pangolin.

During training, none of the models in our article take PANGO lineages as inputs. We use PANGO lineages to cluster the training data into a "training set" and a "validation set". As the reviewer may be aware, this is a standard deep learning practice to split training data, where the "training set" is used to update model parameters and "validation set" is frequently used to check model progress and to stop training when progress has saturated. Conceivably any of the different alternative methods can be used to split sequences into the "training set" and the "validation set" – for example, a random split of the data, or some other clustering method. These methods need not be highly accurate, but simply need to split the data into "training set" and "validation set" such that the sequences in the two sets are different from each other so that it is possible to spot when a model is starting to memorize the training set instead of learning generalizable features that can be applied to another dataset. Given that the method for splitting training data into training and validation sets need not be perfect, and also that the GISAID lineage assignments are highly concordant with lineage reassignments performed with the latest version of Pangolin, we believe our manner of training/validation split to be valid.

Reviewer Comment: *The tables need legends describing the content of the columns.*

Author Response: We have updated the tables following the reviewer's comment.

Reviewer Comment: *The related works section seems to mention many key landmark studies which also use AI-based methods to predict or assess sequences of the virus published in Science and Cell in passing without the usual reference to what the authors do that is different or how their presented work is novel or goes beyond these studies. Rather, a commentary is made on the methodological underpinnings of these works but not how this work supersedes or supplements prior work within the field. This section must be substantially reworked to show what is novel about the presented work and how it is differentiated from prior works in this area.*

Author Response: We have completely revamped this material and absorbed it into Section 1, where it is closely incorporated into the general description and placement of our work. We hope the new descriptions address the reviewer's concerns adequately.

Reviewer Comment: *Code availability: there appears to be no discussion of the code used in this study within the main body of the manuscript. For reproducibility, the code used to generate and train the models and assess the performance relative to other works needs to be placed within GitHub or a similar repository under an open-source license. This is an essential pre-request for publication. A URL is given in the tables that pre-pend the submitted manuscript however that GitHub repo has very little documentation, as such I don't feel the methods are reproducible without better documented code.*

Author Response: This was a misconception on our side that manuscript metadata on Code and Data availability will be automatically inserted into the manuscript by the submission system. We realize that this is not the case. Following this, we have added information on Code and Data availability directly in the manuscript in Section 6. We have also created demo push-button scripts that a user may launch to perform the complete training pipeline for our script. We have added detailed description on code use to our repository.
